# Cooperative Modulation of Mineral Growth by Prismatic-Associated Asprich Sequences and Mg(II)

**DOI:** 10.3390/ijms13033949

**Published:** 2012-03-22

**Authors:** Won Kim, Sebastiano Collino, John Spencer Evans

**Affiliations:** 1 Department of Chemical Engineering, Soongsil University, Seoul 156-743, South Korea; 2 Laboratory for Chemical Physics, New York University, New York, NY 10010, USA; E-Mails: sebastianocollino@gmail.com (S.C.); jse1@nyu.edu (J.S.E.)

**Keywords:** biomineralization, mollusk, calcium carbonate, polypeptide, magnesium

## Abstract

Cooperative effects of magnesium ions and acidic polypeptides originating from a family of proteins known as Asprich (mollusk *Atrina rigida*) were studied. In our previous studies, these two acidic polypeptides were found to be effective in controlling the morphology of the calcium carbonate mineral, the main inorganic constituent of prismatic layer of the mollusk shell. Since these Asprich sequences are believed to contain a putative magnesium binding domain, the morphology-controlling effects were further investigated with the addition of magnesium ions. The mineral morphology was dramatically changed by the combined influence of each polypeptides and the magnesium ions, substantiating the recognized importance of magnesium in the formation of calcium carbonate-based biominerals.

## 1. Introduction

Mollusks construct highly organized structures of exoskeleton in the process of their shell biomineralization. The main inorganic material is calcium carbonate, and typically calcite and aragonite among the calcium carbonate polymorphs are utilized in the controlled morphology [1]. Understanding the exquisite controls in polymorph, morphology, and mesocrystal structure has been important to comprehend the unusual physical properties of mollusk shells [2–4]. The structural controls are generally revealed as the biomacromolecular influences on the nucleation and growth of the inorganic building blocks [2,5]. Also, these have inspired new strategies of materials formation based on the specific interactions between organic additives and inorganic materials [6–8].

In addition to the biomacromolecular controls on calcium carbonate, magnesium ions have been considered as an influential co-factor of the shell biomineralization [1,2,9]. The abundance of Mg ions in the living environment for the mollusk species generated early hypotheses on their involvement in biomineralization. Recently, the roles of the Mg ions were studied in the more defined sets of *in vitro* experiments. The combination of a large amount of Mg ions (Mg/Ca = 2:1) and the biomineral-associated proteins facilitated the formation of precursor amorphous phases that ultimately determined the crystal structure and morphology [10]. At similar conditions (Mg/Ca > 1:1), calcite nucleation studies showed a narrow distribution of crystal size, accompanied by morphological change, probably through controlling the kinetics of crystallization [11]. Also, *in situ* AFM studies showed the roughening and inhibition of calcite growth steps with addition of a fairly large amount of Mg ions (Mg/Ca = 3:5 to 2:1) [12]. Still, the mechanism of magnesium involvement is yet to be fully understood; especially further efforts are necessary to obtain clear understanding of the synergistic effects that would be possibly manifested by the combination of mollusk shell proteins and Mg ions.

**Figure 1:**
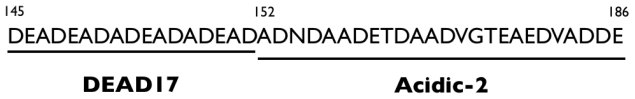
*C*-terminal 42 amino acid domain of Asprich “a” through “g” proteins. The two derived polypeptides (DEAD17, Acidic-2) that comprise this *C*-terminal domain are shown.

Herein, we report the cooperative effects of Mg ions and model polypeptides associated with the mollusk shell protein family, Asprich (*Atrina rigida*) [13–15]. These proteins are unusual in that they possess intrinsically disordered polyanionic sequence domains and/or repetitive sequence blocks. Recently, we found that two sequences derived from highly conserved *C*-terminal domain of the Asprich proteins “a” through “g” (Figure 1; DEAD17, Acidic-2) were more effective in the morphological control of calcite than protein sequences originating from nacre-associated proteins [16–18]. *In situ* AFM observation indicated the ability of these two prismatic sequences to form new step directions [17], although these did not manifest extensively in bulk crystallization probably due to their relative instability [18]. In the present study, a small amount of Mg ions (Mg/Ca = 1:10) was introduced during the bulk crystallization of calcium carbonate in the presence of these two *C*-terminal-derived Asprich sequences to investigate polypeptide-Mg cooperativeness. Also, mechanistic understanding was explored by examining interactions among Mg ions, the model polypeptides, and calcium carbonate (calcite) crystals.

## 2. Results and Discussion

### 2.1. Mineralization of Calcite

**Figure 2:**
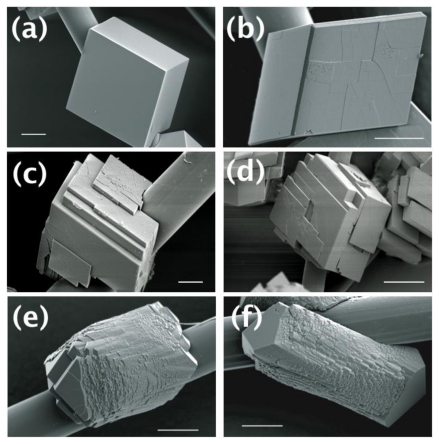
SEM micrographs to show the morphology of the calcite crystals formed (**a**) without any additives; (**b**) with Mg ions; (**c**) with DEAD17; (**d**) with Acidic-2; (**e**) with Mg ions and DEAD17, and (**f**) with Mg ions and Acidic-2. Scalebar = 10 micron.

The calcite crystals were prepared on polyimide fibers using decomposition vapor method (see Experimental Section) under six different conditions: (i) with no additives; (ii) with Mg ions; (iii) with DEAD17; (iv) with Acidic-2; (v) with Mg ions and DEAD17; and (vi) with Mg ions and Acidic-2. Calcite is usually developed as rhombohedral crystals surrounded by {104} faces [11,12,16–18]. This was what we observed without any additive (Figure 2A). When Mg ions were added to the crystallization system (Mg/Ca = 1:10), the overall morphology of the calcite was preserved except that some edges of the calcite crystal showed development of apparently new crystal faces (Figure 2B). When DEAD17 or Acidic-2 was present without Mg, layered structures with the typical {104} faces were observed (Figures 2C,D). Note that the effect of DEAD17 and Acidic-2 on calcite formation without Mg was previously reported [18]. When DEAD17 or Acidic-2 was present in addition to the Mg ions, the calcite morphology was dramatically changed (Figures 2E,F). The crystals showed elongated morphology with the new crystal faces extensively developed in the middle, which were capped by the smooth {104} faces. Also, they possessed a number of micro-domains that appeared near perfectly oriented to each other to form the whole crystals.

**Figure 3:**
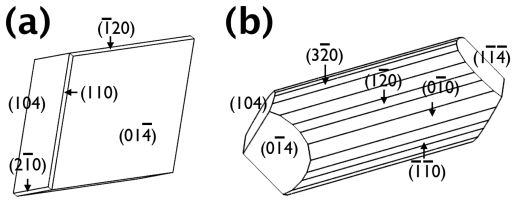
Schematic representations of the morphology of the calcite crystals formed (**a**) with Mg ions and (**b**) with Mg ions and DEAD17. Note that the schematic diagram of (**b**) shows only one of the many micro-domains in Figure 2E.

The morphology of the some crystals was simulated using known crystallographic information of calcite using Shape software [19,20]. The schematic morphological representations of the crystals were shown in Figure 3A (also see Figure 2B) and 3B (also see Figure 2E), for those formed with Mg ions only and with the Mg-DEAD17 combination, respectively. Note that the calcite crystals without any additive and with the Mg-Acidic-2 combination was not shown because the former was already of well known morphology, the latter overall similar to the Mg-DEAD17 case. The morphological analysis approximately identified the newly formed surfaces as {hk0} parallel to the *c*-axis of the calcite crystal. When only Mg was present, emergence of the new faces became slightly visible (Figure 3A). When DEAD17 plus Mg was added, the new faces became considerably developed to make them as the dominant morphological features (Figure 3B). Note that Figure 3B represented only one of the many micro-domains, which assembled to form a whole crystal shown in Figure 2E. The exact shape of each domain was slightly different from each other because of the adjacent domains. Also, more intricate morphology than Figure 3B would be attainable with addition of more {hk0} faces; the simulated morphology of Figure 3B contained only {100}, {110}, and {120}. In summary, we conclude that the Mg ions and Asprich-associated polypeptides worked synergistically to stabilize the new {hk0} faces, parallel to the c-axis of the calcite crystal, to effectively modify the mineral morphology.

Mg has been long recognized as an influential cation associated with the biomineralization of calcium carbonate, and its effects on calcitic morphology have been also studied [9,12,21,22]. In the prior studies, crystals elongated along the c-axis of the calcite were observed, which was similar to the results of the present study. However, noticeable elongation, without any biomacromolecular additives, required Mg ions at least 6–20 times more than in the present study [12,22]. Therefore, the Asprich-associated polypeptides appeared to enhance the ability of Mg to modify calcite morphology when Mg was in low concentrations. Our use of lower Mg content is consistent with known levels of Mg/Ca in rivers [21]. The fact that Mg or polypeptides alone cannot generate these same morphological features indicates the synergistic effects of these species [18].

### 2.2. The Origin of the Morphology Modification

**Figure 4:**
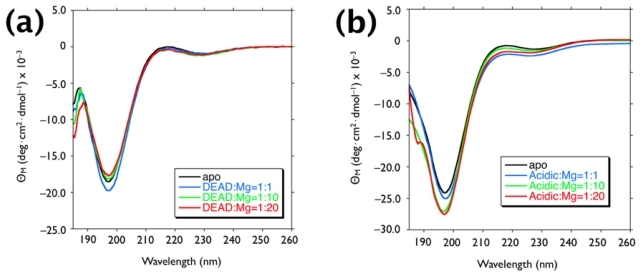
Circular dichroism (CD) spectra of 12 μM (**a**) DEAD17 and (**b**) Acidic-2 in 100 μM Tris-HCl at pH 7.5, in the apo form and in the presence of stoichiometric amounts of MgCl_2_.

To understand the potential synergy between the Mg ions and the Asprich-associated polypeptides, we examined the impact of Mg on the solution conformation of each polypeptide. Circular dichroism experiments were performed with polypeptide/Mg(II) = 1:0, 1:1, 1:10, and 1:20 (Figure 4). As reported in the previous study, DEAD17 and acidic-2 exhibited prominent (–) ellipticity (π-π* transition) at 198 nm with a minor (+) ellipticity band (*n*-π* transition) near 210–220 nm [17,18]. These spectral traits are consistent with unstructured conformation (*i.e.*, random coil). This finding is also consistent with the known structure of Asprich “3”, a member of the Asprich family [14]. When Mg ions are present, no appreciable wavelength shift was observed in the ellipticity bands, indicating that Mg has no detectable influence on the conformation of either peptide.

**Figure 5:**
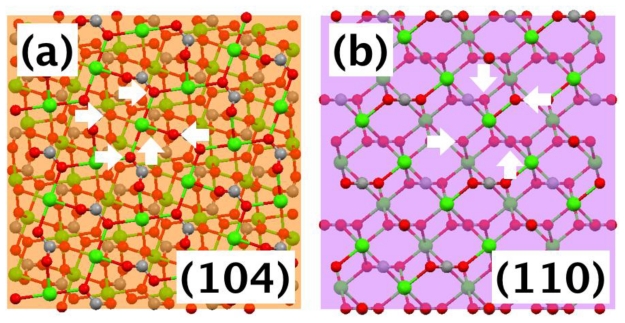
Molecular models showing atomic arrangements of calcite: (**a**) one calcium on (104) plane is surrounded by five oxygen (white arrows) and (**b**) one calcium on (110) plane is surrounded by four oxygen (white arrows). Each plane was shown as a transparent square to show some layers of atoms underneath.

Since Mg ions did not directly induce noticeable changes in the polypeptide conformation, we focused our attention to possible interactions between the polypeptides and the newly emerged {hk0}-type faces with addition of Mg ions, although initially they were of small areas (Figure 2B). The atomic arrangements of the {104}, the typical face of the calcite without any additives, and {hk0} faces were compared. Figure 5 showed the case when {hk0} was {110}. On (104) plane, one calcium was surrounded by five oxygens of carbonate (Figure 5A). Note that within the bulk crystal, one calcium was surrounded by six oxygens. On (110) plane, one calcium was surrounded by four oxygens (Figure 5B). This situation was similar for (120) and (100) planes in that one calcium was surrounded by either four or three oxygens. This analysis indicated that the {hk0} faces were not as well charge balanced as (104). While this made the {hk0} faces less favorable for the equilibrium morphology of calcite, it could provide selective adsorption sites for the anionic polypeptides. Note that recent studies have shown that the Asprich “3” protein can self-assemble and form protein particles within mineralization assays [15]. This implies that DEAD17 and Acidic-2 may form complexes in solution as well, which can act as the agents for mineral interaction.

The surface characteristics can be also compared with the periodic bond chain (PBC) model of Hartman and Perdok [23]. For calcite, {104} is classified to have strongest *F* (flat) character, whereas {110} less strong *F* or *S* (stepped), {100} *S*, and {120} K (kinked) [24,25]. This classification was in good agreement with the morphology observation in Figure 2, where smooth {104} and rough (probably stepped and kinked) {hk0} faces were noticed. While the stepped or kinked characters made these {hk0} faces less favorable to be expressed in the equilibrium morphology, once again they could serve as adsorption sites for the Asprich-associated polypeptides.

We believe that these surface characteristics provide suitable conditions for the negatively charged polypeptides to adsorb and further develop the {hk0} faces, once these surfaces were initially stabilized by Mg ions. Therefore, the synergistic effects of the Mg ions and the Asprich-associated polypeptides seemed to arise from the fact that the Mg ions were able to stabilize the initial {hk0} faces where the Asprich-associated polypeptides could preferentially adsorb over {104} faces. Once polypeptide adsorption starts to slow the growth of the {hk0} faces in the normal direction, these faces become kinetically stable to exhibit the observed mineral morphology. This interpretation can also explain our previous observations, where the extensive morphology modification was not observed with the DEAD17 and Acidic-2 when Mg was not in the bulk mineralization system [18].

The adsorption scenario is also in agreement with the multiple micro-domains observed with the Mg-DEAD17 and Mg-Acidic-2 additions (Figure 2C,D). When the {hk0} faces were kinetically stabilized with the dynamic adsorption of the polypeptides, some surface regions could resume the growth in the normal direction and then the lateral growth. Repetition of this perturbed growth would form the currently observed overall morphology of the multiple micro-domains that appeared near perfectly oriented to each other [26]. In fact, similar situation (so-called “mineral bridges”) was proposed as one of the growth mechanisms of mollusk exoskeleton [27].

Currently, the role of -DE- sequence of the Asprich protein is not fully understood, which has been suggested as potential Mg binding sites based on the sequence comparison with RNA helicases [13]. The negatively charged domains would be certainly effective in the interaction with Ca of various calcite surfaces. Still, we could not entirely preclude the direct involvement of Mg during the adsorption of the polypeptides. Although Mg titration did not appreciably change the peptide conformation, calcite-surface adsorbed Mg can contribute to the preferential binding of the peptides through specific interactions with the -DE- sequence. This possible function would be not incompatible with the proposed adsorption scenario. In fact, it could enhance the selectivity of the morphology modification ability of the polypeptides.

## 3. Experimental Section

### 3.1. *In Vitro* Mineralization and Crystal Analysis

We employed a polyimide (Kevlar) assay for induced growth of calcium carbonate crystals in the presence of Mg ions and the prismatic model polypeptides. The polypeptides were synthesized and purified as described earlier [17,18]. *N*- and/or *C*-termini were capped to simulate peptide bond attachment at the appropriate termini. The prismatic polypeptides are *N*^α^-acetyl/*C*^α^-amide-capped 17 AA DEAD17 and *N*^α^-acetyl-capped/*C*-terminal free 25 AA Acidic-2. Figure 1 shows the AA sequences of each model polypeptide.) For the assay, clean, HCl-treated fibers were submerged in polystyrene Petri dishes containing 3 mL of 10 mM CaCl_2_ and 1 mM MgCl_2_ in deionized distilled water that contains 100 μM polypeptide. Negative control conditions consisted of no added peptide. Note that we had previously reported assays devoid of Mg ions showing effects of the polypeptides [18]. A pinhole opening (1–2 mm) was introduced in each Petri dish cover. Petri dishes were then incubated at 15 °C for 16 h in a sealed chamber (1 L volume) containing 2 g of solid (NH_4_)_2_CO_3_ (decomposition vapor method). After the introduction of vapor, the average pH of assay solutions reaches approximately 8.0–8.3, and thus Asp, Glu residues would be fully deprotonated. At the conclusion of the assay period, Kevlar samples were washed, dried, and prepared for scanning electron microscopy (SEM). SEM imaging was conducted using a Hitachi *S*-3500N scanning electron microscope at 5 kV after thin Au coating. The SEM images presented in this report are representative of 10–20 different crystals in each assay sample. Cropping of SEM images and adjustment of brightness/darkness and contrast levels were performed using Adobe Photoshop. Mercury (version 2.3; The Cambridge Crystallographic Data Centre, Cambridge, UK) and Shape (version 6.0; Shape Software, Kingsport, TN, USA) software was used along with the known crystallographic information to investigate molecular models and crystal morphologies [17]. While small lattice distortions were possible with incorporation of a few percent of Mg in calcite, it was not further considered in the current study [11,28].

### 3.2. Circular Dichroism Spectrometry

Circular dichroism (CD) spectra were obtained for DEAD17 and Acidic-2 at 25 °C, using an AVIV 60 CD spectrometer (60DS software version 4.1t). The CD spectrometer was previously calibrated with *d*-10-camphorsulfonic acid. The peptide samples (apo form) were dissolved and diluted to final concentrations of 12 μM in 100 μM Tris-HCl (pH 7.5). Effects of Mg ions were studied by having 1, 10, and 20-fold amount of MgCl_2_ with respect to the peptide concentration. For all spectra, wavelength scans were conducted from 185 to 260 nm with appropriate background buffer subtraction, using a total of three scans with 1 nm bandwidth and 0.5 nm/s scan rate. In all CD spectra, mean residue ellipticity [θ_M_] is expressed in deg·cm^2^·dmol^−1^.

## 4. Conclusions

In summary, the synergistic effects of Mg ions and Asprich-associated polypeptides were studied for the morphology modification of calcite mineral. The resulting crystals were deviated from the equilibrium {104} rhombohedral morphology, and {hk0} faces were extensively exhibited. This behavior was not seen for the Asprich-associated polypeptides without Mg ions. Also, the small amount of Mg ions (Mg/Ca = 1:10) was not able to exert such effects. Our experiments and analysis suggested that the Mg ions stabilized the {hk0} faces, which became extensively expressed with selective adsorption of the polypeptides. In addition to the comprehensive morphological change, the perturbed growth due to the peptide adsorption caused mesocrystal-like structures by forming a number of micro-domains. Our results provide a new possible process for the proteins to employ an inorganic component, Mg, to enhance their ability of shaping the exoskeleton of the mollusks. This process could be also employed as a useful strategy in the morphology-controlled crystallization of various materials, by allowing formation of conventionally unstable crystal faces.

## References

[1] Lowenstam H.A., Weiner S (1989). On Biomineralization.

[2] Weiner S., Addadi L. (1997). Design strategies in mineralized biological materials. J. Mater. Chem.

[3] Almqvist N., Thomson N.H., Smith B.L., Stucky G.D., Morse D.E., Hansma P.K. (1999). Methods for fabricating and characterizing a new generation of biomimetic materials. Mater. Sci. Eng. C.

[4] Li X.D., Chang W.-C., Chao Y.J., Wang R.Z., Chang M. (2004). Nanoscale structural and mechanical characterization of a natural nanocomposite materials: the shell of red abalone. Nano Lett.

[5] Belcher A.M., Wu X.H., Christensen R.J., Hansma P.K., Stucky G.D., Morse D.E. (1996). Control of crystal phase switching and orientation by soluble mollusc-shell proteins. Nature.

[6] Evans J.S. (2003). “Apples” and “oranges”: comparing the structural aspects of biomineral- and ice-interaction proteins. Curr. Opin. Colloid Interface Sci.

[7] Sarikaya M., Tamerler C., Jen A.K.-Y., Schulten K., Baneyx F. (2003). Molecular biomimetics: nanotechnology through biology. Nat. Mater.

[8] Cölfen H., Antonietti M. (2005). Mesocrystals: Inorganic superstructures made by highly parallel crystallization and controlled alignment. Angew. Chem. Int. Ed.

[9] Lippmann F (1973). Sedimentary Carbonate Minerals.

[10] Raz S., Hamilton P.C., Wilt F.H., Weiner S., Addadi L. (2003). The transient phase of amorphous calcium carbonate in sea urchin larval spicules: the involvement of proteins and magnesium ions in its formation and stabilization. Adv. Funct. Mater.

[11] Han Y.-J., Wysocki L.M., Thanawala M.S., Siegrist T., Aizenberg J. (2005). Template-dependent morphogenesis of oriented calcite crystals in the presence of magnesium ions. Angew. Chem. Int. Ed.

[12] Davis K.J., Dove P.M., Wasylenki L.E., DeYoreo J.J. (2004). Morphological consequences of differential Mg^2+^ incorporation at structurally distinct steps on calcite. Am. Mineral.

[13] Gotliv B.-A., Kessler N., Sumerel J.L., Morse D.E., Tuross N., Addadi L., Weiner S. (2005). Asprich: A novel aspartic acid-rich protein family from the prismatic shell matrix of the bivalve Atrina rigida. Chem. Bio. Chem.

[14] Ndao M., Keene E., Amos F.A., Rewari G., Ponce C.B., Estroff L., Evans J.S. (2010). Intrinsically disordered mollusk shell prismatic protein that modulates calcium carbonate crystal growth. Biomacromolecules.

[15] Ndao M., Ponce C.B., Evans J.S. Evidence of self-association and aggregation-promoting sequences within the “acidic” biomineralization protein, Asprich 3. Biochemistry.

[16] Kim I.W., Darragh M.R., Orme C., Evans J.S. (2006). Molecular “tuning” of crystal growth by nacre-associated polypeptides. Cryst. Growth Des.

[17] Kim I.W., Giocondi J.L., Orme C., Collino S., Evans J.S. (2008). Morphological and kinetic transformation of calcite crystal growth by prismatic-associated Asprich sequences. Cryst. Growth Des.

[18] Collino S., Kim I.W., Evans J.S. (2006). Identification of an “acidic” *C*-terminal mineral modification sequence from the mollusk shell protein Asprich. Cryst. Growth Des.

[19] Graf D.L. (1961). Crystallographic tables for the rhombohedral carbonates. Am. Mineral.

[20] Chung J., Kim I.W. (2011). Oriented crystallization of xanthine derivatives sublimated on self-assembled monolayers. Korean J. Chem. Eng.

[21] Falk R.L. (1974). The natural history of crystalline calcium carbonate: effect of magnesium content and salinity. J. Sediment. Petrol.

[22] DeYoreo J.J., Dove P.M. (2004). Shaping crystals with biomolecules. Science.

[23] Hartman P., Perdok W.G. (1955). On the relations between structure and morphology of crystals. Acta Crystallogr.

[24] Heijnen W.M.M. (1985). The morphology of gel grown calcite (in Russian). N. Jb. Miner. Mh.

[25] Aquilano D., Calleri M., Natoli E., Rubbo M., Sgualdino G. (2000). The {104} cleavage rhombohedron of calcite: theoretical equilibrium properties. Mater. Chem. Phys.

[26] Walton A.G. (1967). The Formation and Properties of Precipitates.

[27] Schäffer T.E., Ionescu-Zanetti C., Proksch R., Fritz M., Walters D.A., Almqvist N., Zaremba C.M., Belcher A.M., Smith B.L., Stucky G.D. (1997). Does abalone nacre form by heteroepitaxial nucleationor by growth through mineral bridges?. Chem. Mater.

[28] Pokroy B., Quintana J.P., Caspi E.N., Berner A., Zolotoyabko E. (2004). Anisotropic lattice distortions in biogenic aragonite. Nat. Mater.

